# Ovarian Somatic Cells: Cellular Heterogeneity and Functional Dynamics Across Health, Ageing, and Disease

**DOI:** 10.3390/cells15201870

**Published:** 2026-10-14

**Authors:** Paola Rebuzzini, Andrea Fantinato, Giulia Brazzale, Giulia Fiorentino, Margherita Isernia, Valeria Merico, Danilo Cimadomo, Maurizio Zuccotti

**Affiliations:** 1 Laboratory of Biology and Biotechnology of Reproduction, Department of Biology and Biotechnology “Lazzaro Spallanzani”, University of Pavia, Via Ferrata 9, 27100 Pavia, Italy; andrea.fantinato01@universitadipavia.it (A.F.); giulia.brazzale01@universitadipavia.it (G.B.); giulia.fiorentino@unipv.it (G.F.); margherita.isernia01@universitadipavia.it (M.I.); valeria.merico@unipv.it (V.M.); danilo.cimadomo@unipv.it (D.C.); 2 IVI Institute, IVI Institute Research Alliance, 00197 Rome, Italy

**Keywords:** ovary, ovarian somatic cells, granulosa cells, theca cells, stroma cells, immune-derived cells, luteal cells, ovarian endothelial cells, ovarian epithelial cells, fertility, ageing, pathology

## Abstract

The ovary is a highly dynamic organ in which the somatic cell population plays essential roles in coordinating folliculogenesis, steroidogenesis, and tissue homeostasis. Increasing evidence highlights substantial cellular heterogeneity and functional plasticity within the ovarian somatic compartment, which is dynamically modulated across the reproductive lifespan. The ovarian somatic cells exhibit distinct transcriptional profiles and functional states that evolve across developmental stages and throughout the reproductive cycle. During ageing, somatic cell composition and function are reshaped, promoting inflammatory responses, stromal remodelling, and reduced support for oocyte developmental competence. Dysregulation of their function and plasticity is also increasingly implicated in reproductive disorders (such as polycystic ovary syndrome, premature ovarian insufficiency and ovarian fibrosis). This review aims to consolidate emerging evidence of the central role of ovarian somatic cells and provides a unifying framework that integrates their roles in physiological ovarian function, ageing, and clinically relevant ovarian disorders.

## 1. Introduction

**Figure 1:**
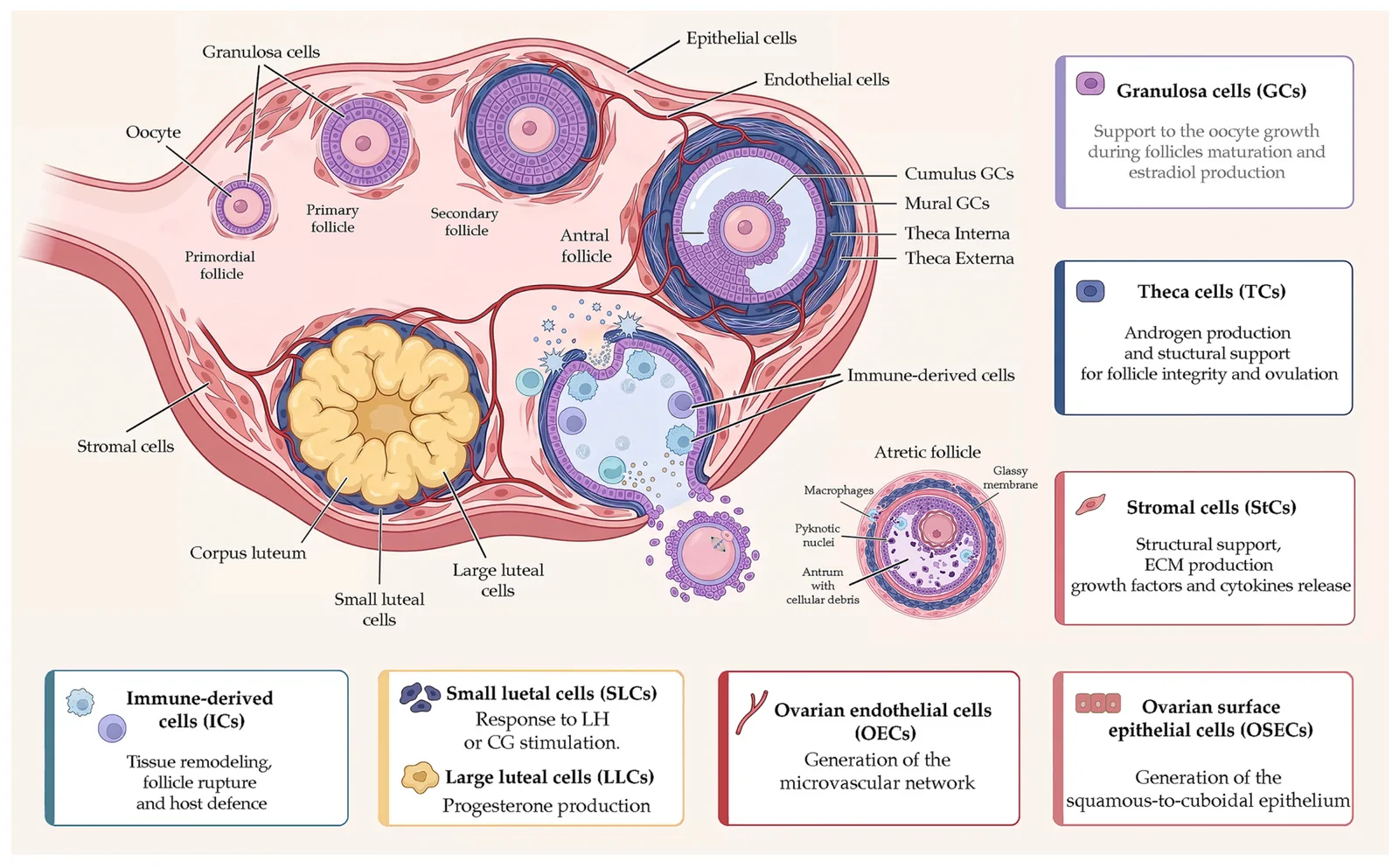
Schematic representation of ovarian folliculogenesis and functional architecture of the follicular niche, with the spatiotemporal progression of follicle maturation, starting from the quiescent primordial follicle pool up to the formation of the corpus luteum. Oocyte maturation is supported by a highly specialised multicellular environment of SCs, with distinct functions. The terminal stage of follicular life is represented by the atretic follicle, characterised by specific hallmark features such as pyknotic nuclei, the formation of a glassy membrane, and macrophage-mediated clearance of cellular debris. The graphic in this figure has been generated with the assistance of Gemini Notebook.

The ovary is a female mammalian organ with both endocrine and reproductive functions [1], where folliculogenesis represents a finely orchestrated process that relies on the coordinated, dynamic and bidirectional interaction between the growing oocytes and several somatic cell (SC) populations, under precise hormonal regulation and a continuous remodelling of the tissue architecture [1,2,3,4]. During folliculogenesis, dormant primordial follicles are progressively recruited, grow through preantral and antral stages or undergo elimination by atresia, and are ultimately selected for ovulation (Figure 1). Primordial follicles consist of a small oocyte arrested in prophase of meiosis I, surrounded by a single layer of flattened somatic pre-granulosa cells. Upon activation, these cells become cuboidal and proliferative, marking the transition to the primary follicle, surrounded by a single layer of granulosa cells (GCs), while the zona pellucida begins to form around the oocyte and the follicle prepares for further growth and differentiation. As development proceeds, the follicle becomes secondary, with GCs proliferating into multiple layers. The theca cell (TC) compartment begins to emerge, followed by the pre-antral stage, in which the follicle enlarges further in the absence of a fluid-filled cavity. The subsequent antral follicle stage is marked by the formation of a fluid-filled antrum, increased hormonal responsiveness, and progressive specialisation of granulosa and TC functions, ultimately leading to selection of the dominant follicle for ovulation [5]. Following this latter, the ruptured follicle undergoes a rapid and coordinated transdifferentiation into the corpus luteum, a transient endocrine gland whose primary function is the high-capacity secretion of progesterone required for implantation and early pregnancy maintenance. This luteinisation process converts GCs and theca cells (TCs) into two major steroidogenic luteal populations: large luteal cells (LLCs), derived predominantly from GCs, and small luteal cells (SLCs), originating mainly from TCs (internal part) [6] (Figure 1).

Beyond follicle-intrinsic somatic cells (SCs), histological and single-cell profiling techniques allowed the identification of additional ovarian somatic populations that contribute to the orderly progression by shaping the local microenvironment, sustaining intercellular crosstalk, and providing the structural, metabolic, and immunological support for the developing follicles. In particular, stromal cells (StCs) help create the structural and biochemical niche required for follicle activation and growth, and immune-derived cells (ICs) modulate local inflammation, tissue remodelling, and the dynamic crosstalk that sustains normal follicular development or elimination and oocyte ovulation. Also, ovarian endothelial cells (OECs) permeate the ovarian parenchyma, forming a dynamic, hormone-responsive microvascular network essential for folliculogenesis, ovulation and luteal function, and ovarian surface epithelial cells (OSECs) give rise to a monolayer squamous-to-cuboidal epithelium that covers and protects the entire female organ [7,8,9,10,11,12,13,14] (Figure 1).

This review is intended to consolidate emerging evidence of the central role of all ovarian SCs in physiological conditions, during ageing, and in some ovarian disorders, highlighting important implications for polycystic ovary syndrome [PCOS; recently suggested to be renamed as polyendocrine metabolic ovarian syndrome (PMOS)], primary ovarian insufficiency (POI) and ovarian fibrosis (OF). This review aims to address a critical gap in the literature by providing a cohesive conceptual framework to synthesise a rapidly expanding, yet highly fragmented, field.

## 2. The Somatic Component of the Mammalian Ovary Under Physiological Conditions

### 2.1. Granulosa Cells

GCs represent the predominant somatic lineage within the ovarian follicle, modulating every stage of oocyte growth, follicular maturation or atresia [8,9,14,15,16] (Figure 1). Initially organised as a single avascular layer of squamous (primordial) or cuboidal (primary) cells around the follicle, GCs undergo progressive dynamic morphological transitions and functional specialisation as folliculogenesis advances. They transition into highly proliferative, multi-layered follicle-stimulating hormone (FSH)-responsive cuboidal cells to later diversify into cumulus cell (CC; proximal to the oocyte) and mural cell (MC; distal to the oocyte) subpopulations in the fully-grown antral follicle [17,18] (Figure 1). Both human and mouse cumulus cells (CCs) and mural cells (MCs) display distinct transcriptional signatures (Table S1) (for human: [19]; for mouse: [20]), and their remarkable transcriptional plasticity allows them to rapidly adjust their functional program in response to hormonal cues, as well as to oocyte-derived factors (e.g., GDF9 and BMP15) and to microenvironmental changes. CCs prioritize metabolic support and oocyte maturation, expressing high levels of glycolytic enzymes (e.g., ALDOA, ENO1, LDH1) and amino acid transporters (e.g., SLC38A3) [21,22,23] (Table S1), whereas MCs assume endocrine dominance, synthesizing estrogens (most of the estradiol) *via* CYP19A1/aromatase-mediated conversion of androgens (androstenedione, testosterone) under FSH stimulation and secrete inhibin A/B and activin to regulate systemic FSH [20] (Table S1). Mammalian granulosa cell (GC) differentiation is mirrored by a dynamic transcriptional signature: GC identity is maintained by the forkhead box protein L2 (FOXL2) transcription factor in both human and mouse [24], while the FSH receptor (FSHR) [25], the aromatase (CYP19A1) [25], anti-Müllerian hormone (AMH) [26], inhibin subunit beta A (INHBA) [27,28], and kit ligand (KITLG) [29] support their endocrine, paracrine, and trophic roles. In addition, intercellular gap junctions together with undocked connexin 43 hemichannels [30] guarantee the metabolic and signalling connection among GCs and with the oocyte in vivo, coupling all the cells metabolically and enabling the fine organisation between the oocyte meiotic progression and the acquisition of its developmental competence [31] (Table S1). Compared with human GCs, mouse GCs proliferate more rapidly, exhibit tighter coupling between FSH responsiveness and *Cyp19a1* induction, and undergo earlier, more sharply defined mural-versus-cumulus specification. By contrast, human GCs display greater transcriptional heterogeneity, rely less on gonadotropin-driven proliferation, and develop within a more protracted follicular growth phase, reflecting species-specific differences in folliculogenesis [20,32].

In a recent single-cell and spatial transcriptomic study, the cellular taxonomy of the GC compartments in the human ovary was substantially refined, evidencing a more considerable heterogeneity and dynamic behaviour than that suggested by conventional cell-type classifications [9]. Notably, three distinct populations of GCs were identified in different areas of the antral follicle. GC subtype 1 (AMH^+^ cells), predominantly located in the cumulus layer, represents an early-stage developmental state characterised by high proliferative capacity (PCNA^high^), active guanosine triphosphate and glucose metabolism and high expression of HSD17B1, a key enzyme required for steroidogenesis. GC subtype 2 (GSTA1^+^ cells), localised in the mural layer of follicles, is enriched for processes related to extracellular matrix (ECM) remodelling and cytokine production; it maintains strong proliferation (PCNA^high^) and plays a central role in cholesterol transport and steroid biosynthetic processes *via* elevated Apolipoprotein E expression. The GC subtype 3 exhibits a widespread distribution across antral follicles, lacks key steroidogenic machinery (HSD17B1^-^/APOE^-^ cells) and displays low proliferative potential (PCNA-^low^). Young and middle-aged human ovaries are enriched in functional, proliferative subtypes 1 and 2, whereas aged ovaries are dominated by subtype 3, highlighting their progressive spatiotemporal transitions [9].

Lastly, in both human and mouse ovaries, GCs are the first cellular compartment that undergoes proliferative arrest during follicular atresia. This latter represents an irreversible physiological process occurring throughout folliculogenesis [33], characterised by hormonally regulated apoptosis and degenerative changes that drive the regression of non-viable follicles. Through this process, ovarian homeostasis is maintained by eliminating the vast majority of follicles (>99%) throughout reproductive life, thereby selecting only the highest-quality oocytes for ovulation [34]. Morphological hallmarks of GC degeneration include intercellular detachment, cytoplasmic shrinkage and vacuolization, nuclear membrane breakdown, chromatin condensation, and pyknosis [35,36]. Interestingly, in antral follicles, GC decay initiates in the mural compartment and progressively extends toward the TC layer, while CC degeneration occurs only at the terminal stages of atresia [35].

### 2.2. Theca Cells

TCs constitute the mesenchymal-derived somatic and vascularized compartment of the ovarian follicle, emerging from the surrounding fibroblast-like stroma during the transition from secondary to antral follicles [37] (Figure 1; Table S1). Although the differentiation hierarchy that generates distinct TC types remains elusive, morphological and multi-omics analyses allowed the identification of TC subtypes each with different structural and functional properties: theca interna (TI) and theca externa (TE) layers (Figure 1) [38,39]. TI cells are highly specialised cells with elevated steroidogenic activity, synthesising androgens under the regulation of luteinizing hormone (LH) [40], subsequently aromatised into estrogens by neighbouring GCs (defined as a two-cell, two-gonadotropin system) [41]. Conversely, the TE layer, composed of fibroblast-like and smooth muscle–like cells and a significant quantity of collagen fibres, confers elasticity to the follicular wall [37,42] (Figure 1; Table S1). These cells, highly vascularized and innervated, modulate biomechanical forces and regulate the exchange of signalling molecules between the follicle and the surrounding stroma. Functionally, by generating contractile forces, TE cells contribute to follicle rupture during ovulation [43], and emerging evidence suggests that the TE cells participate in paracrine communication, modulating GC and TI cells’ activity *via* cytokines (e.g., Interleukin- 6 and 1β, TNFα), growth factors [e.g., TFGβ, BMP4 and 6, EGF, Insulin-like Growth Factor (IGF)] and mechanical triggers [37]. A recent high-resolution transcriptomic mapping unveiled distinct spatial and functional niches within the human ovary, comprising four main theca and stromal subpopulations. While theca and stromal subtypes 1 and 4 populate specific stromal regions in the medulla and cortex of the ovary, subtypes 2 and 5 drive folliculogenesis and ovulation, declining significantly during ovarian ageing. Concurrently, a myofibroblast-like population (subtype 3) contributes to the maintenance of the ovarian vasculature [9].

In addition, consistent with their highly vascularized nature, TCs are particularly susceptible to the vascular remodelling that accompanies follicular atresia [44,45]. The progressive reduction of the thecal capillary network, associated with apoptosis of OECs, is considered an early event in the atretic process. This vascular regression impairs the local delivery of gonadotropins and trophic factors, ultimately compromising TC function and contributing to follicular demise [46]. Key molecular markers of TC identity and function include 17-hydroxylase/17,20-lyase (CYP17A1) and LH/Choriogonadotropin Receptor (LHCGR), specifically for TI cells, and Insulin-like peptide 3 (INSL3), a well-established marker of TC differentiation (Table S1). In both mouse and human ovaries, TCs do not acquire distinct morphological characteristics until the transition from primary to secondary follicles, which occurs approximately one week postnatally and during early childhood, respectively. In mice, commitment to the TC lineage is established much earlier, as neonatal ovaries already harbour a defined population of stem/progenitor cells destined for their formation [5,38,47], while in humans, functional maturation of TCs occurs later and requires rising LH levels. Morphological differentiation of TCs precedes their full endocrine competence and begins at the secondary (preantral) follicle stage [39]. While murine TCs show rapid proliferation and *Cyp17a1* early expression in response to LH, human TCs display a more heterogeneous transcriptional profile and a prolonged maturation timeline, with increased complexity in androgen synthesis and paracrine signalling. Specific pathways, such as Hedgehog, IGF, TGF-β, and PI3K–AKT, modulate TC differentiation and androgen synthesis. Notably, TCs also contribute to the ovarian immune-endocrine interactions, secreting chemokines and cytokines for the recruitment of resident ICs, thereby coordinating tissue remodelling and ovulation-associated inflammation [48].

### 2.3. Stromal Cells

Ovarian StCs constitute a heterogeneous and dynamic mesenchymal compartment that plays a central role in supporting the structural, biochemical and biomechanical properties of the ovary, thereby contributing to follicular organisation, tissue elasticity and ovarian homeostasis (Figure 1; Table S1).

This population comprises fibroblast-like cells, progenitor cells and perivascular cells [49]. In addition, StCs are central regulators of follicle recruitment and growth by producing growth factors [IGF, Vascular Endothelial Growth Factor (VEGF), and FGF], which favour angiogenesis, TC differentiation, and GC–oocyte communication [50]. Molecular markers commonly associated with StCs include vimentin, Platelet-Derived Growth Factor Receptor beta (PDGFRβ), and actin alpha 2 (ACTA2) (Table S1).

StCs exhibit a high transcriptional plasticity [51], as they adapt ECM production in response to hormonal fluctuations, local paracrine cues, and mechanical stress. This plasticity supports the cyclical remodelling of the ovarian cortex and medulla, while also underpinning critical processes such as follicular expansion, ovulation, and luteinisation [35,52]. StCs modulate the biomechanical gradient that characterises the ovarian cortex and medulla through the differential deposition of ECM components, including fibrillar collagens, elastin, proteoglycans, and glycosaminoglycans [3,49]. This spatial heterogeneity generates distinct tissue stiffness profiles, with the cortical region generally exhibiting greater stiffness than the medullary compartment. This biomechanical patterning is critical for ovarian physiology, as mechanical cues derived from the ECM regulate primordial follicle activation and growth through mechanotransduction pathways, including Hippo-YAP/TAZ signalling [3,49]. In addition, StCs actively modulate ovarian tissue mechanics through the secretion of ECM-remodeling enzymes, such as matrix metalloproteinases and lysyl oxidases, which govern collagen turnover, cross-linking, and ECM organisation. These processes dynamically regulate tissue stiffness and facilitate follicle rupture during ovulation [53,54].

### 2.4. Immune-Derived Cells

In Mammals, ICs constitute a resident and dynamic component within the ovary which actively participates in folliculogenesis, ovulation, and luteal function, in addition to their conventional role in host defence. This population includes different types of cells, e.g., macrophages, dendritic cells, natural killer cells, T and B lymphocytes, and innate lymphoid cells. Generally, ICs populate both stromal and follicular niches and, by secreting cytokines, chemokines, and growth factors [55], modulate angiogenesis and ECM remodelling, and the physiological inflammatory cascades required for follicle rupture during ovulation [55,56,57] (Figure 1; Table S1).

Ovarian ICs show pronounced transcriptional plasticity, responding dynamically to hormonal cues (LH, FSH, progesterone, and estrogen stimulation) and local follicular signals (e.g., oocyte-derived GDF9 and BMP15). Their plasticity enables fine-tuning of inflammatory cascades that facilitate follicular rupture and the following corpus luteum formation. The ICs mainly expressed tissue-resident immune cell markers (e.g., CD69 and ITGB2), a variegated gene signature of T-cells (e.g., CD2, CD3G, CD8A) and antigen-presenting cells (e.g., CD14, HLA-DRA, HLA-DQB1, B2M) [52] (Table S1).

In the human ovary, the macrophage compartment exhibits marked heterogeneity, with functional states that change during ageing or with tissue remodelling, from a more immunoregulatory to a pro-inflammatory milieu [8,58,59,60]. Human histological and single-cell studies indicate that macrophages and T lymphocytes are present throughout the whole ovarian cycle, but their abundance and distribution vary according to follicular development, follicular atresia, corpus luteum formation and regression [58,59,61,62]. On the contrary, in rodents’ studies, ovarian IC recruitment, activation, and cytokine production appear to be tightly coordinated and temporally synchronised with the oestrous cycle and ovulatory events [63]. Although the endocrine and inflammatory principles governing follicular rupture and luteal development are broadly conserved, the cellular composition and molecular mediators of ovarian immune responses differ between rodents and humans. These inter-species differences should be considered when interpreting experimental findings. Several ovulatory mediators are shared across species, but their expression profiles or regulatory mechanisms are not necessarily identical. Therefore, rodent data provide an essential mechanistic framework, but cycle-dependent immune findings should not be extrapolated directly to women without confirmation in well-staged human ovarian tissue.

Ultimately, macrophages also play a crucial role in follicular atresia. They mediate the clearance of degenerating follicles by phagocytosing apoptotic GCs and cellular debris, thereby contributing to tissue remodelling and the maintenance of ovarian homeostasis [35].

### 2.5. Luteal Cells

The corpus luteum is composed of two major steroidogenic luteal cell populations, the LLCs and the SLCs (Table S1; Figure 1). LLCs display high constitutive progesterone output and contribute to local hormone secretion. By contrast, SLCs express relatively higher levels of luteinizing LHCGR, thereby exhibiting a more pronounced acute steroidogenic response to LH or chorionic gonadotropin stimulation. Both lineages express the core steroidogenic machinery [including *STAR*, *CYP11A1* (P450scc), and *HSD3B1/2*], but retain distinct transcriptional and functional signatures, memory of their follicular origin. LLCs are enriched in INHBA and PTGS2 (COX-2) proteins and, in several species, express receptors for prostaglandin E2, positioning them as key mediators of both luteal function and luteolysis [64]. SLCs show higher expression of *CYP17A1* and *LHCGR*, and respond acutely to LH *via* the cAMP/PKA pathway to stimulate cholesterol mobilisation and progesterone synthesis [65]. The acquisition of full luteal steroidogenic capacity is tightly coupled to angiogenesis and tissue remodelling. Luteinizing GCs and TCs secrete high levels of Vascular Endothelial Growth Factor A (VEGFA), placental growth factor (PGF), and Endocrine Gland-derived Vascular Endothelial Growth Factor (EG-VEGF)/prokineticin 1 (PROK1), driving the massive neovascularisation.

In the absence of pregnancy, functional luteolysis is initiated by a decline in LH support and, in most nonprimate mammals, by pulsatile uterine PGF_2_α acting through Gq-coupled receptors to activate protein kinase C, inhibit cAMP production, and suppress STAR-mediated cholesterol transport. This is followed by structural luteolysis, characterised by luteal cell apoptosis, ECM remodelling, and infiltration of ICs (particularly macrophages and T lymphocytes). These latter clear cellular debris and amplify local cytokine and prostaglandin signals [66,67]. On the contrary, if pregnancy occurs, embryonic signals, e.g., chorionic gonadotropin, rescue the corpus luteum by sustaining luteotropic support, maintaining angiogenesis, and dampening luteolytic immune and prostaglandin pathways. Through this regulated balance among luteinisation, angiogenesis, and luteolysis, the corpus luteum cells act as the principal endocrine effectors of the post-ovulatory ovary, translating ovulatory signals into a progesterone-dominated environment that determines reproductive success.

### 2.6. Ovarian Endothelial Cells

OECs form the inner lining of blood and lymphatic vessels that go through the ovarian stroma, theca layer, and corpus luteum. The established vascular structure guarantees oxygen, key nutrients, hormones, metabolic precursors and ICs to access follicles and/or luteal tissue and to deliver waste products away from the follicles (Table S1; Figure 1). Recent single-cell and spatial transcriptomic studies of human [52,68] and mouse [10,14] ovaries consistently identify OECs by canonical endothelial markers, e.g., PECAM1 (CD31), CDH5 (VE cadherin), VWF, CLDN5, and CD34, together with signatures of angiogenic activation (e.g., KDR/VEGFR2, NOTCH4), that vary with follicle stage and luteinisation. These cells respond to locally produced VEGFA, PGF, and endocrine gland–derived VEGF (EG-VEGF/PROK1) from GCs, TCs, and luteal cells, translating the endocrine and paracrine cues into changes in permeability, sprouting, and vessel remodelling. During follicle growth, angiogenesis is largely confined to the theca layer and the stroma, but after ovulation, an extensive neovascularisation of the corpus luteum supports its high steroidogenic output, subsequently reduced during luteolysis. OECs actively shape the ovarian microenvironment, linking vascular supply to follicle selection, ovulatory rupture, and luteal lifespan.

### 2.7. Ovarian Surface Epithelial Cells

The ovarian surface epithelium is a highly dynamic tissue that undergoes repeated cycles of ovulation-induced rupture and repair, while retaining a degree of mesothelial plasticity that underlies both its regenerative capacity (Table S1; Figure 1). In both human and mice, OSECs express typical epithelial keratins (KRT7, KRT8, KRT18, KRT19), MUC1, and PAX8, and retain mesenchymal features (e.g., VIM, CDH2), reflecting their mesothelial origin and plasticity. This latter is functionally exploited at each ovulation, when the follicle ruptures through the OSECs, creating a controlled wound [69]. The proliferation of OSECs (mainly LGR5^+^ epithelial progenitors), together with the deposition of the ECM components during ovulatory wound repair, guarantees the surface regeneration and the preservation of the organ integrity [70].

The dynamics of ovarian surface epithelial turnover and the distribution of putative epithelial progenitor cells show important species-specific differences. In mice, the short oestrous cycle (typically 4–5 days) and recurrent ovulation impose repeated episodes of epithelial disruption/repair. Accordingly, LGR5-expressing progenitor cells have been identified within the ovarian surface epithelium and, in some studies, in the ovarian hilar region, where they contribute to epithelial maintenance and repair [71]. In humans, the longer menstrual cycle and the occurrence of a single ovulatory event *per* cycle result in a different temporal organisation of ovarian remodelling. Available evidence indicates that LGR5 expression is trivial in the homeostatic adult ovarian surface epithelium, although rare LGR5-positive cells have been identified in the hilum and in proliferative epithelial areas [72]. These findings underline the importance of considering species-specific differences, and observations from mouse models should not be directly extrapolated to human ovarian epithelial homeostasis without further validation.

## 3. Pathophysiological Aspects of Ovarian Somatic Cells During Ageing and Disease

### 3.1. The Role of SCs in Ovarian Ageing

With advancing age, the mammalian ovary undergoes a progressive decline in oocyte quantity and quality, mainly driven by both germ cell depletion and dysfunction of SC components. While the depletion of the primordial follicle pool has long been considered the primary determinant of reproductive ageing, accumulating evidence suggests that dysfunction of ovarian SCs within the ovarian microenvironment may contribute to reproductive ageing and ovarian disorders, although the relative contribution of SC alterations remains incompletely defined in humans. Beyond their reproductive role, ovarian SCs are increasingly recognised as important regulators of the ageing ovarian microenvironment and of local metabolic adaptation; however, the extent to which these alterations are causal or secondary to follicle depletion remains unresolved [73].

**Figure 2:**
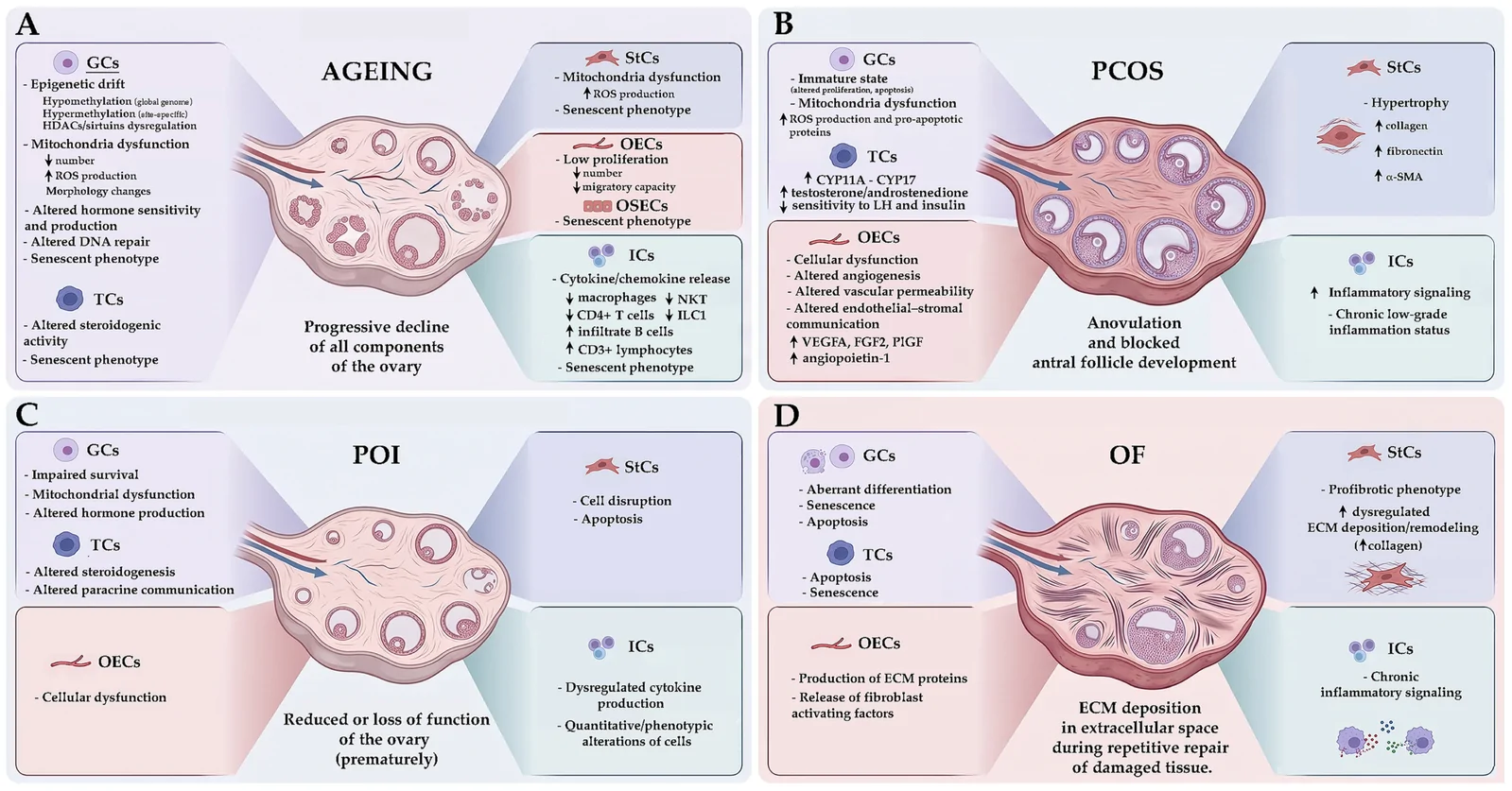
Schematic representation of alterations in the ovarian SC components during (**A**) ageing and in three ovarian pathologies, i.e., (**B**) PCOS, (**C**) POI and (**D**) OF. In the aged ovary, the functionality of several SC types is compromised, determining the decline of the entire organ. In PCOS and POI, ovarian dysfunction is primarily caused by a defect in the GCs and TCs. In OF, StCs create a pro-fibrotic milieu, and macrophages represent the major contributing immune population, determining a chronic inflammatory state in the tissue. The graphic of this figure has been generated with the assistance of Gemini Notebook.

Several biological dysfunctions contribute to the decline in SC performance that accompanies reproductive senescence and fertility decline (Figure 2) [74,75,76].

#### 3.1.1. Epigenetic Drift

Epigenetic drift represents a gradual, age-dependent erosion of DNA methylation and histone modification patterns. Age-associated alterations in DNA methylation, histone modifications, and non-coding RNA expression have been reported in ovarian tissues and ovarian SC models. These changes are consistent with an age-related epigenetic drift, although the available evidence is not yet uniform across species, ovarian cell types, or experimental settings. Epigenetic drift has been evidenced mainly in human ovarian SCs and, specifically, in MCs. In aged ovaries, human MCs, isolated from pooled follicles immediately after oocyte retrieval, exhibit global hypomethylation accompanied by site-specific hypermethylation at promoters of genes involved in follicular development and DNA repair [77]. Epimutations and age-related differentially methylated regions, exceeding those in other non-ovarian SCs, indicate a unique methylation signature in the follicular somatic compartment. These changes involve genes like *AMH*, *FSHR*, *ZFP57*, and *VTRNA2-1,* as well as genes associated with mitochondrial dynamics and antioxidant defence. These epigenetic alterations have been associated with changes in the expression of genes involved in gonadotropin responsiveness, steroidogenesis, mitochondrial function, and antioxidant defence. Functionally, GCs from human aged ovaries may exhibit reduced gonadotropin responsiveness, impaired steroidogenic activity, and increased oxidative stress; however, the causal contribution of individual epimutations remains to be determined [77,78]. Also, human aged GCs show increased levels of repressive marks (e.g., H3K27me3), associated with transcriptional silencing of genes required for follicle survival and cellular metabolism [79]. Altered histone deacetylase and sirtuin activities in human and mouse GCs, including changes in SIRT1 and SIRT6, have been reported in experimental models of ovarian ageing and have been linked to mitochondrial dysfunction and senescence-related phenotypes [80,81,82,83] (Figure 2A). To the best of our knowledge, direct evidence for epigenetic drift in ovarian SC populations other than GCs remains limited. This represents a major knowledge gap, particularly because recent single-cell and spatial transcriptomic studies indicate that ageing affects multiple ovarian cell types and is accompanied by extensive stromal remodelling and immune-cell (IC) alterations. Future age-stratified studies based on purified cell populations and integrating single-cell epigenomics with spatial multi-omics will be essential to determine whether other ovarian SCs undergo cell type-specific changes in DNA methylation, chromatin accessibility, histone modifications, and non-coding RNA expression.

#### 3.1.2. Mitochondrial Dysfunction and Oxidative Stress

Age-related mitochondrial alterations include decreased oxidative phosphorylation efficiency, accumulation of mitochondrial DNA mutations, and impaired mitophagy. These changes lead to reduced ATP production and increased generation of reactive oxygen species (ROS).

Mitochondrial alterations have been reported in ovarian SCs during ageing, particularly in GCs, and may represent an early component of the ageing-associated ovarian phenotype [84]. These latter are particularly susceptible to oxidative stress due to their high metabolic activity and reliance on mitochondrial function. Mitochondrial dysfunction in human GCs from periovulatory follicles is a central mechanism driving ovarian ageing and reproductive decline, together with a reduction in their number in old females [85]. Impaired oxidative phosphorylation is the main cause of human MC dysfunction [86]. Using various cellular models, it has been demonstrated that human and mouse GCs exposed to elevated ROS levels show increased levels of DNA damage (single- and double-strand breaks) [87] and decreased DNA repair capability [88]. Oxidative stress also contributes to intercellular signalling dysregulation within the follicle. ROS-mediated modifications of signalling pathways can alter GC proliferation, differentiation, and survival in pre-ovulatory follicles of mice, thereby disrupting the finely tuned mechanisms governing folliculogenesis [89]. Although these findings provide mechanistic insights into the effects of oxidative stress on follicular function, their relevance to human folliculogenesis requires further validation, given potential species-specific differences in GC responses. GCs and CCs normally exchange healthy mitochondria *via* tunnelling nanotubes; using in vitro co-culture systems of human ovarian SCs, it has been shown that with age this process is significantly reduced, impairing their highly coordinated metabolisms (Figure 2A) [90].

Finally, elevated ROS levels promote the onset of cellular senescence in GCs and StCs, and in turn, senescent cells exacerbate oxidative stress through altered metabolic activity and inflammatory signalling (Figure 2A).

#### 3.1.3. Cellular Senescence and Metabolic Changes

Cellular senescence represents a state of irreversible cell cycle arrest accompanied by profound metabolic and secretory changes. Ageing is associated with reduced proliferative capacity and senescence-related changes in several ovarian SC populations, together with reduced responsiveness to hormones and impaired steroidogenic capacity. However, the magnitude, timing, and cellular distribution of these alterations vary among the different cell types [91].

A reduction in GC abundance and morphological alterations have been reported in aged ovaries, particularly in animal models and in selected human studies; their morphology changes, becoming elongated, swollen, and with cytoplasmic vacuolation [85,92,93]. Senescent GCs undergo metabolic alterations, shifting from glycolysis and pyruvate/NADPH production to reduced aerobic glycolysis and mitochondrial respiration, a transition that significantly reduces the fundamental metabolic support provided to oocytes (particularly oxidative phosphorylation) for their maturation and developmental competence (Figure 2A) [94].

Although less documented, both StCs and TCs exhibit age-related dysfunction through specific pathways, e.g., estrogen signalling, insulin pathways, and circadian rhythms. Given their role in maintaining the ovarian ECM and stromal architecture, age-associated dysfunction of StCs may compromise tissue integrity. The presence of senescence-associated β-galactosidase in StCs and TCs earlier than in GCs and CCs may accelerate oocyte ageing and, in general, ovarian decay. Aged StCs contribute to tissue fibrosis through ECM remodelling, which in turn reduces tissue elasticity and impairs follicular vascularisation [95]. In parallel, senescent or functionally aged TCs exhibit altered steroidogenic activity in vivo, which can compromise androgen production and thereby affect downstream estrogen synthesis required to sustain follicular function (Figure 2A) [96].

During ovarian ageing, the relative quantitation of ICs undergoes important modifications. In aged mice, changes in the ovarian IC composition have been reported, including reductions in selected macrophage, type 1 innate lymphoid cell, natural killer T cell, and naïve CD4+ T-cell populations, indicating a decline in immune competence in the ovary [63,97]. Increased accumulation of CD3+ lymphocytes has been observed in ovarian tissue in some ageing models and may be associated with inflammatory remodelling and reduced follicular reserve [98], as well as B cells, which infiltrate the tissue, contributing to the pro-inflammatory environment. The accumulation or activation of immune cells, together with the release of pro-inflammatory and pro-fibrotic mediators and senescence-associated secretory phenotype (SASP) factors, may contribute to a chronic inflammatory microenvironment and progressive remodelling of ovarian tissue. Also, using a mouse in vivo model, it has been demonstrated that during ageing the capability of ICs to eliminate senescent cells diminishes, resulting in their accumulation [99] (Figure 2A). Because these findings derive primarily from animal studies, their relevance to human ovarian ageing remains to be established [97].

In old ovaries, OECs lose their proliferative potential, reducing their number and migratory capacity, leading to decreased capillary density in the ovarian stroma and the inner lining of developing follicles (theca layer). Reduced vascular support may compromise the delivery of oxygen, nutrients, and circulating endocrine signals to the follicular microenvironment [100,101,102]. Similarly, OSECs undergo structural and molecular shifts during ovarian ageing, including cellular senescence and decreased expression of postovulatory repair markers (Figure 2A). The cyclical “tear-and-repair” process imposes mechanical and biochemical stress on OSECs. At each ovulation, proteolytic degradation of the ovarian epithelium generates a focal wound that is accompanied by a localised inflammatory response, with the release of cytokines and ROS. The efficiency of the protective mechanisms in OSECs declines with age and cumulative ovulatory burden [103,104]. These observations support a model in which repeated ovulatory injury may contribute to epithelial stress and senescence-related changes over time, although the relative contribution of ovulatory burden compared with endocrine, inflammatory, and intrinsic ageing processes remains uncertain.

### 3.2. The Role of SCs in Ovarian Disorders

Beyond their established role in physiological ovarian function and age-related fertility decline, ovarian SCs are now recognised as active drivers of ovarian pathology. Dysregulation of SC signalling, metabolism, and identity lies at the core of several reproductive disorders, with particularly profound consequences for follicular health and ovarian impairment. Among the spectrum of ovarian diseases, we focus on PCOS, POI, and OF, three conditions in which SC dysfunction is not merely a bystander but a central pathogenic node that amplifies follicular failure and accelerates reproductive decline.

#### 3.2.1. Polycystic Ovary Syndrome

PCOS is the most common hormonal disorder affecting about 8–13% of women during the reproductive age. The pathology causes anovulation, and it is characterised by blocked antral follicle development before the preovulatory stage [105].

GC dysfunction is strongly associated with PCOS onset and development, as their altered proliferation, apoptosis, oxidative stress, and mitochondrial dysfunction contribute to follicular arrest and impaired oocyte maturation. Moreover, PCOS-associated hyperandrogenism, characterised by elevated testosterone and androstenedione levels, and altered FSH signalling disrupt GC proliferation and differentiation, thereby contributing to follicular dysfunction and impaired folliculogenesis. As a result, GCs remain in an immature proliferative state rather than undergoing proper differentiation, thereby altering normal follicular development (Figure 2B) [105,106,107].

In addition, GCs display dysregulated apoptosis, showing both increased apoptosis and apoptosis resistance, depending on the stage of follicle development. Since PCOS ovaries have many small antral follicles that fail to mature, GCs frequently show increased expression of pro-apoptotic markers (e.g., BAX, Forkhead box O3 and caspases). This event contributes to their arrest and poor dominant follicle selection. However, GCs from PCOS patients may become relatively resistant to apoptosis under certain hormonal conditions (especially hyperandrogenism and insulin resistance), allowing abnormal follicles to persist instead of undergoing normal atresia [106,107,108]. GCs present high levels of oxidative stress and impaired mitochondrial energy production due to mitochondrial dysfunction, as a consequence of chronic low-grade inflammation and hyperandrogenism (Figure 2B) [109].

PCOS is also linked to TC dysfunction through increased androgen biosynthesis and altered sensitivity to hormonal signals, such as LH and insulin. The upregulation in TCs of the enzymes involved in androgen production (CYP11A and CYP17) promotes hyperandrogenism, disrupting physiological folliculogenesis [110,111]. Ovarian StC dysfunction mediates the increased stromal volume and stromal hypertrophy, marked by enhanced collagen deposition, increased α-smooth muscle actin expression, and elevated fibronectin and TGF-β–related remodelling, and ICs, such as macrophages, B and T cells, promote inflammatory signalling and a chronic low-grade inflammatory status (Figure 2B) [95]. Finally, OECs may also contribute to PCOS pathophysiology through altered angiogenesis, vascular permeability, and endothelial–stromal communication. In women with PCOS, increased stromal and thecal vascularity, together with altered concentrations of angiogenic mediators, has been reported [112]. In particular, increased VEGFA levels in serum and follicular fluid, as well as altered VEGF bioavailability, have been described in selected PCOS human cohorts. Elevated FGF2 and placental growth factor (PlGF), as well as altered follicular-fluid concentrations of PDGF-BB, PDGF-DD, and angiopoietin-1, further support the presence of an imbalanced angiogenic milieu (Figure 2B) [113,114,115,116]. Nevertheless, current findings should be interpreted cautiously, as most human data measuring angiogenic factors are derived from blood or follicular fluid rather than directly characterising endothelial cells within ovarian tissue.

#### 3.2.2. Primary Ovarian Insufficiency

POI is a heterogeneous clinical syndrome that occurs in 1–2% of women, in which the ovaries reduce or stop functioning normally before age 40, causing irregular periods, low estrogen levels and impaired fertility [117].

As reported for PCOS pathology, GC and TC dysfunction has been implicated in POI pathophysiology, although the strength of evidence differs across POI etiologies and experimental models. Impaired GC survival, defective mitochondrial function, and abnormal steroidogenesis have been associated with impaired follicular survival and may contribute to accelerated follicular loss [118,119,120]. Similarly, TC alterations disrupt steroidogenesis and paracrine communication within the follicle, leading to impaired GC function, increased follicular apoptosis, and premature depletion of ovarian activity (Figure 2C) [120].

POI is characterised by activation of proinflammatory pathways, secondary to estrogen deficiency, implicating the ovarian immune compartment as a key mediator of the disease. Quantitative and phenotypic alterations in IC populations (macrophages, T and B lymphocytes, and antigen-presenting cells) were detected in peripheral blood serum of POI-affected patients, disrupting cellular immunity and precipitating inflammatory injury [121,122]. Dysregulated cytokine production, encompassing IL-1α, IL-20, IL-17, IL-2, IL-8, IL-6, TNF-α, IFN-γ and IL-21 detected in follicular fluid or serum of POI-affected patients, establishes a sustained proinflammatory milieu that promotes follicular depletion (Figure 2C) [123,124].

The contribution of other ovarian SCs to POI remains insufficiently defined. Even though direct evidence in humans remains limited, it has been proposed that disruption of human ovarian StCs might contribute to the damage of the structural and paracrine environment required for follicular maintenance and ovarian homeostasis, thereby promoting follicular loss (Figure 2C) [95]. Also, young women with POI showed significant ovarian and systemic OEC dysfunction compared with age-matched women with normal ovarian function, as a consequence of sex-steroid deficiency [125].

#### 3.2.3. Ovarian Fibrosis

OF is a severe pathological condition characterised by excessive fibroblast proliferation, myofibroblast differentiation, and uncontrolled deposition of ECM in the extracellular space during the process of repetitive repair of damaged tissue. This altered ovarian condition leads to infertility and various endocrine disorders [126,127,128].

Fibrotic ovarian remodelling reflects a multicellular process driven by altered crosstalk among several ovarian SC populations. StCs are increasingly recognised as central organisers of the fibrotic response. They acquire a profibrotic phenotype, characterised by enhanced collagen deposition, dysregulated matrix remodelling, and reduced tissue compliance, thereby contributing to stromal stiffening and progressive niche dysfunction (Figure 2D). This remodelling is likely to impair follicular support and amplify microenvironmental disruption, establishing a self-reinforcing cycle in which fibrosis and follicular decline exacerbate one another [95]. Also, GC function is compromised; the coordinated communication appears to weaken, favouring altered matrix reorganisation and a shift toward a profibrotic niche. In particular, GC apoptosis, senescence, and/or aberrant differentiation may reduce trophic signalling and disrupt the balance between ECM deposition and its degradation, thereby contributing to ECM element accumulation and tissue stiffening (Figure 2D) [128]. These changes are likely to reinforce follicular atresia and impair the structural integrity of the ovarian cortex, creating a feed-forward loop in which GC dysfunction and fibrosis mutually exacerbate ovarian dysfunction. Being TCs closely linked to both GC function and stromal homeostasis, their dysfunction augments a feed-forward loop of fibrotic remodelling and progressive decline in ovarian function [95]. In parallel, ICs (mainly macrophages) appear to amplify fibrosis through chronic inflammatory signalling and matrix-modifying pathways, linking immune activation to progressive stromal stiffening (Figure 2D) [95,128]. Histological studies, including Picrosirius Red-based analyses, consistently document age-associated increases in fibrillar collagen deposition and disorganisation of collagen architecture, supporting the notion that OF is accompanied by a measurable rise in tissue rigidity [126,129,130]. Finally, OECs also promote fibrosis; in response to TGF-β levels, OECs produce ECM proteins or secrete fibroblast-activating factors. Endothelin, a potent vasoconstrictor polypeptide produced and secreted by OECs, indirectly stimulates fibrogenesis and the expression of pro-fibrotic genes and proteins (Figure 2D) [131,132].

## 4. Discussion and Conclusions

The ovarian somatic compartment constitutes a highly specialised and dynamic cellular network that governs folliculogenesis, endocrine function, and tissue homeostasis through tightly coordinated interactions with the germline component. Beyond providing structural and metabolic support, SCs actively shape the ovarian microenvironment across the reproductive lifespan, thereby sustaining follicle developmental competence and reproductive potential. Accumulating evidence indicates that age-associated remodelling of SC populations, together with alterations in their signalling and metabolic programs, plays a central role in the progressive decline of ovarian reserve and function. Aberrant SC behaviour has emerged as a key determinant of ovarian pathophysiology, contributing to inflammatory, fibrotic and degenerative processes.

Although recent studies have elucidated key somatic drivers in PCOS, POI, and OF, translating these findings requires addressing several technical bottlenecks: the limited availability of human ovarian tissue, the reliance on in vitro and non-human animal models, the lack of spatial context in single-cell transcriptomic analyses, and the challenge of establishing causality within self-reinforcing processes such as somatic senescence and tissue fibrosis. Deciphering the molecular identity, functional plasticity, and intercellular communication networks of ovarian SCs is crucial to deepening our understanding of the cellular principles underlying ovarian physiology, ageing, and pathology. Furthermore, such knowledge could also shed light on regenerative capabilities that could be exploited to devise innovative therapeutic strategies. In this context, a deeper mechanistic understanding of ovarian SC biology could ultimately provide insights into approaches aimed at restoring ovarian homeostasis, preserving reproductive potential, and improving women’s reproductive health. Indeed, the identification of ovarian SCs as active drivers and modulators of disease raises the possibility of developing compartment-oriented therapeutic strategies. In PCOS and other conditions characterised by GC metabolic dysfunction, interventions might aim at restoring mitochondrial quality control, limiting oxidative stress, and improving mitophagy, which might help preserve GC viability and follicular support. In parallel, the selective elimination of senescent SCs or the pharmacological modulation of the SASP could attenuate the inflammatory and profibrotic signalling associated with ovarian ageing and POI. Also, targeting the stromal extracellular matrix remodelling-driven profibrotic axis, aberrant collagen deposition, and altered tissue mechanics might provide an additional strategy to preserve follicular architecture and ovarian function. Finally, modulation of the ovarian immune microenvironment, including the rebalancing of macrophage and other IC phenotypes, may further contribute to restoring tissue homeostasis. However, although these approaches remain largely supported by in vitro and animal studies, their translation to the clinic is, to date, limited by the cellular heterogeneity of the ovary, the difficulty of achieving cell- and compartment-specific delivery, and the risk of disrupting physiological follicular remodelling or immune regulation.

## Data Availability

No new data were created or analyzed in this study. Data sharing is not applicable to this article.

## Supplementary Materials

The following supporting information can be downloaded at: https://www.mdpi.com/article/10.3390/cells15201870/s1, Table S1: Ovarian somatic cell types: localization, functions and cellular markers.

## Abbreviations

The following abbreviations are used in this manuscript:

SC	somatic cell
GCs	granulosa cells
TC	theca cell
TCs	theca cells
LLCs	large luteal cells
SLCs	small luteal cells
SCs	somatic cells
StCs	stromal cells
ICs	immune-derived cells
OECs	ovarian endothelial cells
OSECs	ovarian surface epithelial cells
PCOS	polycystic ovary syndrome
PMOS	polyendocrine metabolic ovarian syndrome
POI	primary ovarian insufficiency
OF	ovarian fibrosis
FSH	follicle-stimulating hormone
CC	cumulus cell
MC	mural cell
CCs	cumulus cells
MCs	mural cells
GC	granulosa cell
FOXL2	forkhead box protein L2
FSHR	follicle-stimulating hormone receptor
AMH	anti-Müllerian hormone
INHBA	inhibin subunit beta A
KITLG	kit ligand
ECM	extracellular matrix
TI	theca interna
TE	theca externa
LH	luteinizing hormone
IGF	Insulin-like Growth Factor
LHCGR	Luteinizing Hormone/Choriogonadotropin Receptor
VEGF	Vascular Endothelial Growth Factor
INSL3	Insulin-like peptide 3
VEGFA	Vascular Endothelial Growth Factor A
PGF	placental growth factor
PROK1	prokineticin 1
EG-VEGF	Endocrine Gland-derived Vascular Endothelial Growth Factor
PDGFRβ	Platelet-Derived Growth Factor Receptor beta
ACTA2	actin alpha 2
IC	immune-cell
ROS	reactive oxygen species
SASP	senescence-associated secretory phenotype

## References

[1] Richards J.S., Pangas S.A. (2010). New insights into ovarian function. Handb. Exp. Pharmacol..

[2] Baerwald A.R., Adams G.P., Pierson R.A. (2012). Ovarian antral folliculogenesis during the human menstrual cycle: A review. Hum. Reprod. Update.

[3] Fiorentino G., Cimadomo D., Innocenti F., Soscia D., Vaiarelli A., Ubaldi F.M., Gennarelli G., Garagna S., Rienzi L., Zuccotti M. (2023). Biomechanical forces and signals operating in the ovary during folliculogenesis and their dysregulation: Implications for fertility. Hum. Reprod. Update.

[4] Zhang Q., Sun F., Zhang R., Zhao D., Zhu R., Cheng X., Long X., Hou X., Yan R., Cao Y. (2024). The evolution of ovarian somatic cells characterized by transcriptome and chromatin accessibility across rodents, monkeys, and humans. Life Med..

[5] Edson M.A., Nagaraja A.K., Matzuk M.M. (2009). The mammalian ovary from genesis to revelation. Endocr. Rev..

[6] Przygrodzka E., Hou X., Zhang P., Plewes M.R., Franco R., Davis J.S. (2021). PKA and AMPK Signaling Pathways Differentially Regulate Luteal Steroidogenesis. Endocrinology.

[7] Zhang X., Cao Q., Rajachandran S., Grow E.J., Evans M., Chen H. (2023). Dissecting mammalian reproduction with spatial transcriptomics. Hum. Reprod. Update.

[8] Jones A.S.K., Hannum D.F., Machlin J.H., Tan A., Ma Q., Ulrich N.D., Shen Y.C., Ciarelli M., Padmanabhan V., Marsh E.E. (2024). Cellular atlas of the human ovary using morphologically guided spatial transcriptomics and single-cell sequencing. Sci. Adv..

[9] Wu M., Tang W., Chen Y., Xue L., Dai J., Li Y., Zhu X., Wu C., Xiong J., Zhang J. (2024). Spatiotemporal transcriptomic changes of human ovarian aging and the regulatory role of FOXP1. Nat. Aging.

[10] Huang R., Kratka C.E., Pea J., McCann C., Nelson J., Bryan J.P., Zhou L.T., Russo D.D., Zaniker-Gomez E.J., Gandhi A.H. (2025). Single-cell and spatiotemporal profile of ovulation in the mouse ovary. PLoS Biol..

[11] Zhao Z.H., Meng T.G., Chen X.Y., Gao F., Schatten H., Ou X.H., Sun Q.Y. (2025). Spatiotemporal and single-cell atlases to dissect regional specific cell types of the developing ovary. Commun. Biol..

[12] Rooda I., Méar L., Hassan J., Damdimopoulou P. (2025). The adult ovary at single cell resolution: An expert review. Am. J. Obstet. Gynecol..

[13] Martinez A.S., Gibson T.J., McKey J. (2025). Single-cell-scale spatial transcriptome of the developing and adult mouse ovary. bioRxiv.

[14] Ren X., Lv T., Liu N., Shi C., Fan J., Zhao N., Kang Q., Wang D. (2026). A unified spatial transcriptome profiling of ten mouse organs. Sci. Data.

[15] Schütz L.F., Batalha I.M. (2024). Granulosa Cells: Central Regulators of Female Fertility. Endocrines.

[16] Guo J., Hu Y., Cao Q., Zhang Y., Jia Y., Liu L., Zeng Y., Wu X., Song Y., Yang M. (2025). GADD45A is Essential for Granulosa Cells Differentiation and Ovarian Reserve in Human and Mice. J. Cell. Mol. Med..

[17] Dhar A., Dockery P., O W.S., Turner K., Lenton E.A., Cooke I.D. (1996). The human ovarian granulosa cell: A stereological approach. J. Anat..

[18] Mork L., Maatouk D.M., McMahon J.A., Guo J.J., Zhang P., McMahon A.P., Capel B. (2012). Temporal differences in granulosa cell specification in the ovary reflect distinct follicle fates in mice. Biol. Reprod..

[19] Grøndahl M.L., Andersen C.Y., Bogstad J., Borgbo T., Boujida V.H., Borup R. (2012). Specific genes are selectively expressed between cumulus and granulosa cells from individual human pre-ovulatory follicles. Mol. Hum. Reprod..

[20] Wigglesworth K., Lee K.B., Emori C., Sugiura K., Eppig J.J. (2015). Transcriptomic diversification of developing cumulus and mural granulosa cells in mouse ovarian follicles. Biol. Reprod..

[21] Sugiura K., Eppig J.J. (2005). Society for Reproductive Biology Founders’ Lecture 2005. Control of metabolic cooperativity between oocytes and their companion granulosa cells by mouse oocytes. Reprod. Fertil. Dev..

[22] Su Y.Q., Sugiura K., Eppig J.J. (2009). Mouse oocyte control of granulosa cell development and function: Paracrine regulation of cumulus cell metabolism. Semin. Reprod. Med..

[23] Rohn M.C.H., Simeone J.M., Doctorman S., Ge P., Hernandez A., Das S., Spann M., Dow M., Segars J., Singh B. (2025). The Functional Role of Cumulus Cells and Their Influence on Oocyte Quality: A Systematic Review. Reprod. Sci..

[24] Pisarska M.D., Barlow G., Kuo F.T. (2011). Minireview: Roles of the forkhead transcription factor FOXL2 in granulosa cell biology and pathology. Endocrinology.

[25] Luo W., Wiltbank M.C. (2006). Distinct regulation by steroids of messenger RNAs for FSHR and CYP19A1 in bovine granulosa cells. Biol. Reprod..

[26] Pellatt L., Rice S., Dilaver N., Heshri A., Galea R., Brincat M., Brown K., Simpson E.R., Mason H.D. (2011). Anti-Müllerian hormone reduces follicle sensitivity to follicle-stimulating hormone in human granulosa cells. Fertil. Steril..

[27] Knight P.G., Glister C. (2001). Potential local regulatory functions of inhibins, activins and follistatin in the ovary. Reproduction.

[28] Findlay J.K., Drummond A.E., Dyson M., Baillie A.J., Robertson D.M., Ethier J.F. (2001). Production and actions of inhibin and activin during folliculogenesis in the rat. Mol. Cell. Endocrinol..

[29] Hutt K.J., McLaughlin E.A., Holland M.K. (2006). Kit ligand and c-Kit have diverse roles during mammalian oogenesis and folliculogenesis. Mol. Hum. Reprod..

[30] Tong D., Li T.Y., Naus K.E., Bai D., Kidder G.M. (2007). In vivo analysis of undocked connexin43 gap junction hemichannels in ovarian granulosa cells. J. Cell Sci..

[31] Ackert C.L., Gittens J.E., O’Brien M.J., Eppig J.J., Kidder G.M. (2001). Intercellular communication via connexin43 gap junctions is required for ovarian folliculogenesis in the mouse. Dev. Biol..

[32] Nakakita B., Mizuta K., Katou Y., Tasaki H., Nakamura T., Yabuta Y., Iwatani C., Tsuchiya H., Kawamoto I., Tsukiyama T. (2026). Evolutionarily divergent transcriptomic programs in ovarian folliculogenesis across mice, monkeys and humans. Development.

[33] Meng L., Jan S.Z., Hamer G., van Pelt A.M., van der Stelt I., Keijer J., Teerds K.J. (2018). Preantral follicular atresia occurs mainly through autophagy, while antral follicles degenerate mostly through apoptosis. Biol. Reprod..

[34] Matsuda F., Inoue N., Manabe N., Ohkura S. (2012). Follicular growth and atresia in mammalian ovaries: Regulation by survival and death of granulosa cells. J. Reprod. Dev..

[35] Wei F., Fan X., Del Valle J.S., Asseler J.D., van der Meeren L.E., Cheng H., Roelen B.A.J., Louwe L.A., Pilgram G.S.K., van der Westerlaken L.A.J. (2023). Classification of Atretic Small Antral Follicles in the Human Ovary. Int. J. Mol. Sci..

[36] Regan S.L.P., Knight P.G., Yovich J.L., Leung Y., Arfuso F., Dharmarajan A. (2018). Granulosa Cell Apoptosis in the Ovarian Follicle-A Changing View. Front. Endocrinol..

[37] Young J.M., McNeilly A.S. (2010). Theca: The forgotten cell of the ovarian follicle. Reproduction.

[38] Liu C., Peng J., Matzuk M.M., Yao H.H. (2015). Lineage specification of ovarian theca cells requires multicellular interactions via oocyte and granulosa cells. Nat. Commun..

[39] Guahmich N.L., Man L., Wang J., Arazi L., Kallinos E., Topper-Kroog A., Grullon G., Zhang K., Stewart J., Schatz-Siemers N. (2023). Human theca arises from ovarian stroma and is comprised of three discrete subtypes. Commun. Biol..

[40] Gilling-Smith C., Willis D.S., Franks S. (1997). Oestradiol feedback stimulation of androgen biosynthesis by human theca cells. Hum. Reprod..

[41] Ryan K.J. (1979). Granulosa-thecal cell interaction in ovarian steroidogenesis. J. Steroid Biochem..

[42] Lind A.K., Weijdegård B., Dahm-Kähler P., Mölne J., Sundfeldt K., Brännström M. (2006). Collagens in the human ovary and their changes in the perifollicular stroma during ovulation. Acta Obstet. Gynecol. Scand..

[43] Thomas C. (2025). Ovulation: A cellular symphony in three movements. Semin. Cell Dev. Biol..

[44] Jiang J.Y., Macchiarelli G., Tsang B.K., Sato E. (2003). Capillary angiogenesis and degeneration in bovine ovarian antral follicles. Reproduction.

[45] Fraser H.M. (2006). Regulation of the ovarian follicular vasculature. Reprod. Biol. Endocrinol..

[46] Acosta T.J., Miyamoto A. (2004). Vascular control of ovarian function: Ovulation, corpus luteum formation and regression. Anim. Reprod. Sci..

[47] Honda A., Hirose M., Hara K., Matoba S., Inoue K., Miki H., Hiura H., Kanatsu-Shinohara M., Kanai Y., Kono T. (2007). Isolation, characterization, and in vitro and in vivo differentiation of putative thecal stem cells. Proc. Natl. Acad. Sci. USA.

[48] Duffy S., Day E.K., Bryson G., Millan D., Lindsay R. (2018). Malignant tumours arising within mature cystic teratomas of the ovary: Prevalence and clinical outcomes. Eur. J. Obstet. Gynecol. Reprod. Biol..

[49] Kinnear H.M., Tomaszewski C.E., Chang A.L., Moravek M.B., Xu M., Padmanabhan V., Shikanov A. (2020). The ovarian stroma as a new frontier. Reproduction.

[50] Pour F.K., Gale Dari M.A., Ramazii M., Keivan M., Farzaneh M. (2024). Mesenchymal Stem Cells-Conditioned Medium; An Effective Cell-Free Therapeutic Option for in vitro Maturation of Oocytes. Curr. Stem Cell Res. Ther..

[51] Wamaitha S.E., Nie X., Pandolfi E.C., Wang X., Yang Y., Stukenborg J.B., Cairns B.R., Guo J., Clark A.T. (2023). Single-cell analysis of the developing human ovary defines distinct insights into ovarian somatic and germline progenitors. Dev. Cell.

[52] Wagner M., Yoshihara M., Douagi I., Damdimopoulos A., Panula S., Petropoulos S., Lu H., Pettersson K., Palm K., Katayama S. (2020). Single-cell analysis of human ovarian cortex identifies distinct cell populations but no oogonial stem cells. Nat. Commun..

[53] Curry T.E., Smith M.F. (2006). Impact of extracellular matrix remodeling on ovulation and the folliculo-luteal transition. Semin. Reprod. Med..

[54] McCord L.A., Li F., Rosewell K.L., Brännström M., Curry T.E. (2012). Ovarian expression and regulation of the stromelysins during the periovulatory period in the human and the rat. Biol. Reprod..

[55] Dai M., Xu Y., Gong G., Zhang Y. (2023). Roles of immune microenvironment in the female reproductive maintenance and regulation: Novel insights into the crosstalk of immune cells. Front. Immunol..

[56] Wu R., Van der Hoek K.H., Ryan N.K., Norman R.J., Robker R.L. (2004). Macrophage contributions to ovarian function. Hum. Reprod. Update.

[57] Ono Y., Nagai M., Yoshino O., Koga K., Nawaz A., Hatta H., Nishizono H., Izumi G., Nakashima A., Imura J. (2018). CD11c+ M1-like macrophages (MΦs) but not CD206+ M2-like MΦ are involved in folliculogenesis in mice ovary. Sci. Rep..

[58] Katabuchi H., Suenaga Y., Fukumatsu Y., Okamura H. (1997). Distribution and fine structure of macrophages in the human ovary during the menstrual cycle, pregnancy and menopause. Endocr. J..

[59] Yang X., Gilman-Sachs A., Kwak-Kim J. (2019). Ovarian and endometrial immunity during the ovarian cycle. J. Reprod. Immunol..

[60] Zhou C., Guo Q., Lin J., Wang M., Zeng Z., Li Y., Li X., Xiang Y., Liang Q., Liu J. (2024). Single-Cell Atlas of Human Ovaries Reveals The Role Of The Pyroptotic Macrophage in Ovarian Aging. Adv. Sci..

[61] Zhang Z., Huang L., Brayboy L., Birrer M. (2025). Single-cell analysis of ovarian myeloid cells identifies age-associated changes in macrophages and signaling dynamics. Biol. Reprod..

[62] Richards O., Cotterell M., Thornton C.A., Rees A. (2026). Revealing the immune landscape of menstrual blood: Unlocking insights into activation, exhaustion, and mitochondrial mass for reproductive health. Immunohorizons.

[63] Ben Yaakov T., Wasserman T., Aknin E., Savir Y. (2023). Single-cell analysis of the aged ovarian immune system reveals a shift towards adaptive immunity and attenuated cell function. Elife.

[64] Mlyczyńska E., Kieżun M., Kurowska P., Dawid M., Pich K., Respekta N., Daudon M., Rytelewska E., Dobrzyń K., Kamińska B. (2022). New Aspects of Corpus Luteum Regulation in Physiological and Pathological Conditions: Involvement of Adipokines and Neuropeptides. Cells.

[65] Miyamoto A., Shirasuna K., Shimizu T., Bollwein H., Schams D. (2010). Regulation of corpus luteum development and maintenance: Specific roles of angiogenesis and action of prostaglandin F2alpha. Soc. Reprod. Fertil. Suppl..

[66] Bachelot A., Binart N. (2005). Corpus luteum development: Lessons from genetic models in mice. Curr. Top. Dev. Biol..

[67] Sugino N., Okuda K. (2007). Species-related differences in the mechanism of apoptosis during structural luteolysis. J. Reprod. Dev..

[68] Fan X., Bialecka M., Moustakas I., Lam E., Torrens-Juaneda V., Borggreven N.V., Trouw L., Louwe L.A., Pilgram G.S.K., Mei H. (2019). Single-cell reconstruction of follicular remodeling in the human adult ovary. Nat. Commun..

[69] Kruk P.A., Auersperg N. (1992). Human ovarian surface epithelial cells are capable of physically restructuring extracellular matrix. Am. J. Obstet. Gynecol..

[70] Singavarapu R., Buchinsky N., Cheon D.J., Orsulic S. (2010). Whole ovary immunohistochemistry for monitoring cell proliferation and ovulatory wound repair in the mouse. Reprod. Biol. Endocrinol..

[71] Ng A., Tan S., Singh G., Rizk P., Swathi Y., Tan T.Z., Huang R.Y., Leushacke M., Barker N. (2014). Lgr5 marks stem/progenitor cells in ovary and tubal epithelia. Nat. Cell Biol..

[72] Kim H., Lee D.H., Park E., Myung J.K., Park J.H., Kim D.I., Kim S.I., Lee M., Kim Y., Park C.M. (2022). Differential epithelial and stromal LGR5 expression in ovarian carcinogenesis. Sci. Rep..

[73] Lu H., Jing Y., Zhang C., Ma S., Zhang W., Huang D., Zhang B., Zuo Y., Qin Y., Liu G.H. (2024). Aging hallmarks of the primate ovary revealed by spatiotemporal transcriptomics. Protein Cell.

[74] Zhu F.Y., Wang L.L., Meng T.G., Wang R.L., Yang Z.X., Cao Y., Zhu G.Y., Jin Z., Gao L.L., Zeng W.T. (2021). Inhibiting bridge integrator 2 phosphorylation leads to improved oocyte quality, ovarian health and fertility in aging and after chemotherapy in mice. Nat. Aging.

[75] Ju W., Zhao Y., Yu Y., Zhao S., Xiang S., Lian F. (2024). Mechanisms of mitochondrial dysfunction in ovarian aging and potential interventions. Front. Endocrinol..

[76] Mani S., Srivastava V., Shandilya C., Kaushik A., Singh K.K. (2024). Mitochondria: The epigenetic regulators of ovarian aging and longevity. Front. Endocrinol..

[77] Olsen K.W., Castillo-Fernandez J., Zedeler A., Freiesleben N.C., Bungum M., Chan A.C., Cardona A., Perry J.R.B., Skouby S.O., Borup R. (2020). A distinctive epigenetic ageing profile in human granulosa cells. Hum. Reprod..

[78] Voros C., Varthaliti A., Mavrogianni D., Athanasiou D., Athanasiou A., Athanasiou A., Papahliou A.M., Zografos C.G., Topalis V., Kondili P. (2025). Epigenetic Alterations in Ovarian Function and Their Impact on Assisted Reproductive Technologies: A Systematic Review. Biomedicines.

[79] Olsen K.W., Castillo-Fernandez J., Chan A.C., la Cour Freiesleben N., Zedeler A., Bungum M., Cardona A., Perry J.R.B., Skouby S.O., Hoffmann E.R. (2021). Identification of a unique epigenetic profile in women with diminished ovarian reserve. Fertil. Steril..

[80] Valerio D., Luddi A., De Leo V., Labella D., Longobardi S., Piomboni P. (2018). SA1/SA2 cohesion proteins and SIRT1-NAD+ deacetylase modulate telomere homeostasis in cumulus cells and are eligible biomarkers of ovarian aging. Hum. Reprod..

[81] Kong D., Yao G., Bai Y., Yang G., Xu Z., Kong Y., Fan H., He Q., Sun Y. (2020). Expression of sirtuins in ovarian follicles of postnatal mice. Mol. Reprod. Dev..

[82] Bao S., Yin T., Liu S. (2024). Ovarian aging: Energy metabolism of oocytes. J. Ovarian Res..

[83] Kapur A., Reddy Y., Rajagopal N., Nayak S., D’Souza N.V., Das I., Sundara B.K. (2026). Decoding Infertility: The Epigenetic Influence of HDACs on Reproductive Function. Reprod. Sci..

[84] Chiang J.L., Shukla P., Pagidas K., Ahmed N.S., Karri S., Gunn D.D., Hurd W.W., Singh K.K. (2020). Mitochondria in Ovarian Aging and Reproductive Longevity. Ageing Res. Rev..

[85] Tatone C., Carbone M.C., Falone S., Aimola P., Giardinelli A., Caserta D., Marci R., Pandolfi A., Ragnelli A.M., Amicarelli F. (2006). Age-dependent changes in the expression of superoxide dismutases and catalase are associated with ultrastructural modifications in human granulosa cells. Mol. Hum. Reprod..

[86] Liu Y., Han M., Li X., Wang H., Ma M., Zhang S., Guo Y., Wang S., Wang Y., Duan N. (2017). Age-related changes in the mitochondria of human mural granulosa cells. Hum. Reprod..

[87] Lin L., Gao W., Chen Y., Li T., Sha C., Chen L., Yang M., Wei H., Chen Y., Zhu X. (2022). Reactive oxygen species-induced SIAH1 promotes granulosa cells’ senescence in premature ovarian failure. J. Cell. Mol. Med..

[88] Talibova G., Bilmez Y., Tire B., Ozturk S. (2024). The DNA double-strand break repair proteins γH2AX, RAD51, BRCA1, RPA70, KU80, and XRCC4 exhibit follicle-specific expression differences in the postnatal mouse ovaries from early to older ages. J. Assist. Reprod. Genet..

[89] Liu X.M., Chen F., Zhang F., Zhao J.Z. (2023). Chk1/2 inhibitor AZD7762 blocks the growth of preantral follicles by inducing apoptosis, suppressing proliferation, and interfering with the cell cycle in granulosa cells. Histol. Histopathol..

[90] Li C.J., Lin L.T., Lin P.H., Sheu J.J., Wen Z.H., Tsui K.H. (2025). Reprogramming the aging ovarian microenvironment via mitochondrial sharing and structural remodeling. Theranostics.

[91] Tang W., Wang K., Feng Y., Tsui K.H., Singh K.K., Stout M.B., Wang S., Wu M. (2025). Exploration of the mechanism and therapy of ovarian aging by targeting cellular senescence. Life Med..

[92] de Bruin J.P., Dorland M., Spek E.R., Posthuma G., van Haaften M., Looman C.W., Te Velde E.R. (2004). Age-related changes in the ultrastructure of the resting follicle pool in human ovaries. Biol. Reprod..

[93] Tatone C., Amicarelli F., Carbone M.C., Monteleone P., Caserta D., Marci R., Artini P.G., Piomboni P., Focarelli R. (2008). Cellular and molecular aspects of ovarian follicle ageing. Hum. Reprod. Update.

[94] Kobayashi H., Yoshimoto C., Matsubara S., Shigetomi H., Imanaka S. (2024). Altered Energy Metabolism, Mitochondrial Dysfunction, and Redox Imbalance Influencing Reproductive Performance in Granulosa Cells and Oocyte During Aging. Reprod. Sci..

[95] Shen L., Liu J., Luo A., Wang S. (2023). The stromal microenvironment and ovarian aging: Mechanisms and therapeutic opportunities. J. Ovarian Res..

[96] Wu H., Zhang Z., Huang Q., Liu B., Liu L. (2026). Theca cells: Active orchestrators of ovarian aging. Reprod. Biol. Endocrinol..

[97] Isola J.V.V., Hense J.D., Osório C.A.P., Biswas S., Alberola-Ila J., Ocañas S.R., Schneider A., Stout M.B. (2024). Reproductive Ageing: Inflammation, immune cells, and cellular senescence in the aging ovary. Reproduction.

[98] Jasti S., Warren B.D., McGinnis L.K., Kinsey W.H., Petroff B.K., Petroff M.G. (2012). The autoimmune regulator prevents premature reproductive senescence in female mice. Biol. Reprod..

[99] Ovadya Y., Landsberger T., Leins H., Vadai E., Gal H., Biran A., Yosef R., Sagiv A., Agrawal A., Shapira A. (2018). Impaired immune surveillance accelerates accumulation of senescent cells and aging. Nat. Commun..

[100] Robinson R.S., Woad K.J., Hammond A.J., Laird M., Hunter M.G., Mann G.E. (2009). Angiogenesis and vascular function in the ovary. Reproduction.

[101] Wang G., Yang R., Zhang H. (2025). Ovarian vascular aging: A hidden driver of mid-age female fertility decline. npj Aging.

[102] Mu L., Wang G., Yang X., Liang J., Tong H., Li L., Geng K., Bo Y., Hu X., Yang R. (2025). Physiological premature aging of ovarian blood vessels leads to decline in fertility in middle-aged mice. Nat. Commun..

[103] Clow O.L., Hurst P.R., Fleming J.S. (2002). Changes in the mouse ovarian surface epithelium with age and ovulation number. Mol. Cell. Endocrinol..

[104] Mara J.N., Zhou L.T., Larmore M., Johnson B., Ayiku R., Amargant F., Pritchard M.T., Duncan F.E. (2020). Ovulation and ovarian wound healing are impaired with advanced reproductive age. Aging.

[105] Franks S., Stark J., Hardy K. (2008). Follicle dynamics and anovulation in polycystic ovary syndrome. Hum. Reprod. Update.

[106] Das M., Djahanbakhch O., Hacihanefioglu B., Saridogan E., Ikram M., Ghali L., Raveendran M., Storey A. (2008). Granulosa cell survival and proliferation are altered in polycystic ovary syndrome. J. Clin. Endocrinol. Metab..

[107] Deng Y., Li H., Song Y., Cen J., Zhang Y., Sui Y., Cui D., Li T.C., Xu Y., Wang C.C. (2022). Whole Genome Transcriptomic Analysis of Ovary Granulosa Cells Revealed an Anti-Apoptosis Regulatory Gene DLGAP5 in Polycystic Ovary Syndrome. Front. Endocrinol..

[108] Chauvin S. (2025). Role of Granulosa Cell Dysfunction in Women Infertility Associated with Polycystic Ovary Syndrome and Obesity. Biomolecules.

[109] Gao Y., Zou Y., Wu G., Zheng L. (2023). Oxidative stress and mitochondrial dysfunction of granulosa cells in polycystic ovarian syndrome. Front. Med..

[110] Kyei-Mensah A.A., LinTan S., Zaidi J., Jacobs H.S. (1998). Relationship of ovarian stromal volume to serum androgen concentrations in patients with polycystic ovary syndrome. Hum. Reprod..

[111] Nelson V.L., Legro R.S., Strauss J.F., McAllister J.M. (1999). Augmented androgen production is a stable steroidogenic phenotype of propagated theca cells from polycystic ovaries. Mol. Endocrinol..

[112] Di Pietro M., Pascuali N., Parborell F., Abramovich D. (2018). Ovarian angiogenesis in polycystic ovary syndrome. Reproduction.

[113] Artini P.G., Monti M., Matteucci C., Valentino V., Cristello F., Genazzani A.R. (2006). Vascular endothelial growth factor and basic fibroblast growth factor in polycystic ovary syndrome during controlled ovarian hyperstimulation. Gynecol. Endocrinol..

[114] Tal R., Seifer D.B., Grazi R.V., Malter H.E. (2013). Angiopoietin-1 and angiopoietin-2 are altered in polycystic ovarian syndrome (PCOS) during controlled ovarian stimulation. Vasc. Cell.

[115] Tal R., Seifer D.B., Arici A. (2015). The emerging role of angiogenic factor dysregulation in the pathogenesis of polycystic ovarian syndrome. Semin. Reprod. Med..

[116] Bao L., Syed R., Aloahd M.S. (2019). Analysis of VEGF gene polymorphisms and serum VEGF protein levels contribution in polycystic ovary syndrome of patients. Mol. Biol. Rep..

[117] Chon S.J., Umair Z., Yoon M.S. (2021). Premature Ovarian Insufficiency: Past, Present, and Future. Front. Cell Dev. Biol..

[118] Tiosano D., Mears J.A., Buchner D.A. (2019). Mitochondrial Dysfunction in Primary Ovarian Insufficiency. Endocrinology.

[119] Houeis L., Donnez J., Dolmans M.M. (2025). Premature Ovarian Insufficiency and Diminished Ovarian Reserve: From Diagnosis to Current Management and Treatment. J. Clin. Med..

[120] Li K., Wen Y., Cui Z., Ling L., Wang C., Zhang B., Liu T. (2025). High-Fat and High-Sugar Diet Induces Murine Premature Ovarian Failure by Promoting Mitochondrial Oxidative Phosphorylation Response. Food Sci. Nutr..

[121] Nelson L.M., Kimzey L.M., Merriam G.R., Fleisher T.A. (1991). Increased peripheral T lymphocyte activation in patients with karyotypically normal spontaneous premature ovarian failure. Fertil. Steril..

[122] Zhang C., Yu D., Mei Y., Liu S., Shao H., Sun Q., Lu Q., Hu J., Gu H. (2023). Single-cell RNA sequencing of peripheral blood reveals immune cell dysfunction in premature ovarian insufficiency. Front. Endocrinol..

[123] Liu P., Zhang X., Hu J., Cui L., Zhao S., Jiao X., Qin Y. (2020). Dysregulated cytokine profile associated with biochemical premature ovarian insufficiency. Am. J. Reprod. Immunol..

[124] Kunicki M., Rzewuska N., Gross-Kępińska K. (2024). Immunophenotypic profiles and inflammatory markers in Premature Ovarian Insufficiency. J. Reprod. Immunol..

[125] Kalantaridou S.N., Naka K.K., Bechlioulis A., Makrigiannakis A., Michalis L., Chrousos G.P. (2006). Premature ovarian failure, endothelial dysfunction and estrogen-progestogen replacement. Trends Endocrinol. Metab..

[126] Briley S.M., Jasti S., McCracken J.M., Hornick J.E., Fegley B., Pritchard M.T., Duncan F.E. (2016). Reproductive age-associated fibrosis in the stroma of the mammalian ovary. Reproduction.

[127] Gu M., Wang Y., Yu Y. (2024). Ovarian fibrosis: Molecular mechanisms and potential therapeutic targets. J. Ovarian Res..

[128] Edepli B.G., Yaba A. (2026). Molecular mechanisms of ovarian fibrosis. Mol. Hum. Reprod..

[129] Landry D.A., Yakubovich E., Cook D.P., Fasih S., Upham J., Vanderhyden B.C. (2022). Metformin prevents age-associated ovarian fibrosis by modulating the immune landscape in female mice. Sci. Adv..

[130] Aitken C.J., Kadhim L., Murray A., Landry D.A. (2026). Ovarian Fibrosis Through Comparative Analysis of Collagen Architecture. Mol. Reprod. Dev..

[131] Wermuth P.J., Li Z., Mendoza F.A., Jimenez S.A. (2016). Stimulation of Transforming Growth Factor-β1-Induced Endothelial-To-Mesenchymal Transition and Tissue Fibrosis by Endothelin-1 (ET-1): A Novel Profibrotic Effect of ET-1. PLoS ONE.

[132] Frangogiannis N. (2020). Transforming growth factor-β in tissue fibrosis. J. Exp. Med..

