# Supplementary material for: Ovarian Somatic Cells: Cellular Heterogeneity and Functional Dynamics Across Health, Ageing, and Disease
Source: Cells. 2026 Oct 14;15(20):1870. doi: 10.3390/cells15201870 (PMC13703262; doi:10.3390/cells15201870)
Supplement: Supplementary file 1 [file cells-15-01870-s001.zip › cells-4529551-supplementary.pdf]

Table S1. Ovarian somatic cell types: localization, functions and cellular markers.

Cell type	Location	Main functions	Human markers		Mouse markers	
			Well-established core	Enriched or state-dependent	Well-established core	Enriched or state-dependent
**Granulosa cells (GCs) – General both subpopulations**	Surround the oocyte within ovarian follicles (multiple layers in antral follicles)	- Support oocyte growth<br>- Estradiol production<br>- Secrete inhibins/activins<br>- Regulate systemic FSH levels<br>- Regulate follicle selection<br>- Luteinize after ovulation<br>- Maintain oocyte meiotic arrest	-FOXL2 *(lineage)*<br>- FSHR<br>- CYP19A1<br>- AMH<br>- WT1<br>- GJA1 (Cx43)<br>- KITLG<br>- CCND2	- AMH *(higher in small antral follicles, declines in pre-ovulatory follicle)*<br>- CYP19A1, FSHR *(increase with follicle growth and FSH dependence)*<br>- WT1 *(high in early/pre-antral stages)*<br>- KITLG *(modulated by oocyte-derived factors)*	- FOXL2 *(lineage)*<br>- FSHR<br>- CYP19A1<br>- AMH<br>- WT1<br>- GJA1<br>- KITL<br>- CCND2	- AMH *(small follicles)*<br>- CYP19A1, FSHR *(antral, FSH-dependent follicles)*<br>- WT1 *(high in early/pre-antral stages)*<br>- KITLG *(modulated by oocyte-derived factors)*
Cumulus cells (*GCs subtypes*)	Wrap the oocyte, form the cumulus-oocyte complex (COC)	- Form direct gap junctions with the oocyte for nutrient/signal exchange<br>- Transport the oocyte through the oviduct<br>- Secrete factors for meiotic arrest<br>- Participate in oocyte maturation and embryonic quality	- GJA1<br>- KITLG<br>- AMH (*only small antral follicles)*<br>- FOXL2<br>- FSHR *(shared with GCs)*	- PTX3, HAS2, GREM1, PTGS2, TNFAIP6 (TSG6), EGFR, CD44, INHBA, BTC *(oocyte competence / cumulus expansion markers; induced by GDF9/BMP15)*	- GJA1<br>- KITL<br>- AMH<br>- FOXL2<br>- FSHR	- PTX3, HAS2, GREM1, PTGS2, TNFAIP6, EGFR, CD44, INHBA *(cumulus expansion and oocyte competence markers)*
Mural cells (*GCs subtypes*)	Form the antral follicle wall, attached to the basal membrane, distant from the oocyte	- Produce estradiol<br>- Secrete inhibin A/B and activin<br>- Mediate FSH signaling for follicle selection<br>- Luteinize after ovulation<br>- Regulate follicular atresia	- FSHR<br>- CYP19A1<br>- AMH (*small–mid antral)*<br>- WT1<br>- FOXL2<br>- GJA1<br>- CCND2<br>- INHBA<br>- INHBB	- FSHR, CYP19A1 (*very high in large, FSH-dependent antral follicles)*<br>- AMH *(high in small antral, decreases with growth)*<br>- NR5A2<br>- NPPC, MRO *(preovulatory MCs)*	- FSHR<br>- CYP19A1<br>- AMH<br>- WT1<br>- FOXL2<br>- GJA1<br>- CCND2<br>- INHBA<br>- INHBB	- FSHR, CYP19A1 *(high in antral follicles)*<br>- AMH *(small antral)*<br>- NR5A2, NPPC, MRO *(MCs in antral/preovulatory follicles)*
**Theca cells (TCs) – General both subpopulations**	Form the follicular coat surrounding granulosa cells	- Produce androgens for estradiol synthesis<br>- Provide structural support and ECM	- CYP17A1<br>- LHCGR<br>- INSL3<br>- STAR	- CYP17A1, LHCGR, STAR *(higher in steroidogenically active theca of growing antral follicles)*	- CYP17A1<br>- LHCGR<br>- INSL3<br>- STAR	- CYP17A1, LHCGR, STAR *(steroidogenic theca of antral follicles)*<br>- ACTA2 *(TE/smooth muscle)*

		- Contribute to follicle integrity and ovulation	- HSD3B1<br>- ACTA2<br>- CYP11A1<br>- NR5A1<br>- COL1A1<br>- VIM	- ACTA2 (*more prominent in TE/smooth muscle-like component)*	- HSD3B1<br>- ACTA2<br>- CYP11A1<br>- NR5A1<br>- COL1A1<br>- VIM	
Theca interna (TI - TCs subtypes)	Directly adjacent to the granulosa layer, separated by the basal membrane	- Produce androgens (androstenedione, testosterone)<br>- Respond to LH signaling<br>- Provide steroid substrates for estradiol production in GCs<br>- Differentiate into theca-lutein cells after ovulation	- CYP17A1 (very high)<br>- LHCGR (high)<br>- STAR (high)<br>- HSD3B1<br>- CYP11A1<br>- NR5A1<br>- INSL3	- CYP17A1, LHCGR, STAR (highest in steroidogenically active antral follicles)	- CYP17A1<br>- LHCGR<br>- STAR<br>- HSD3B1<br>- CYP11A1<br>- NR5A1<br>- INSL3	- CYP17A1, LHCGR, STAR *(antral, steroidogenic theca)*
Theca externa *(TE - TCs subtypes)*	Outer layer of theca, fibrous layer surrounding theca interna, adjacent to ovarian stroma	- Provide structural support via ECM and collagen<br>- Form fibrous capsule around the follicle<br>- Contain smooth muscle–like cells for follicle contraction during ovulation<br>- Contribute to vascular support	- ACTA2 (very high)<br>- TAGLN<br>- MYH11<br>- COL1A1, COL3A1<br>- LAMA2<br>- LAMB1<br>- VIM	- ACTA2, TAGLN, MYH11 (very high in smooth muscle–like cells of TE)<br>- MFAP5, RGS5, EDNRB, CNN1, ACTG2 (smooth muscle / pericyte-like signatures)	- ACTA2<br>- TAGLN<br>- MYH11<br>- COL1A1<br>- COL3A1<br>- LAMA2<br>- LAMB1<br>- VIM<br>- PDGFRB	- ACTA2, TAGLN, MYH11 *(smooth muscle layer)*<br>- MFAP5, RGS5, EDNRB, CNN1, ACTG2
**Stromal cells (StCs)**	Ovarian interstitium (stroma), surrounding follicles, corpus luteum, and blood vessels; form the bulk of the ovarian cortex and medulla	- Provide ECM and structural support<br>- Secrete growth factors and cytokines<br>- Contribute to stroma remodeling<br>- Participate in ovarian repair after ovulation or injury	- VIM<br>- PDGFRA<br>- PDGFRB<br>- FAP<br>- S100A4 (FSP1)<br>-COL1A1<br>- COL3A1<br>- ACTA2 *(myofibroblast subset)*<br>- THY1 *(CD90; fibroblast marker)*	- ACTA2, TAGLN *(myofibroblast / activated stromal states)*<br>- CD24 (*mesenchymal progenitor-like stromal subset; regenerative contexts)*<br>- CXCL12, IGF1, WNT *(paracrine support; context-dependent)*	- VIM<br>- PDGFRA<br>- PDGFRB<br>- FAP<br>- S100A4<br>- COL1A1<br>- COL3A1<br>- ACTA2<br>- THY1	- ACTA2, TAGLN *(activated / myofibroblastic stroma)*<br>- CD24 (*mesenchymal progenitor cells in developing/ emodeling ovary)*<br>- CXCL12, IGF1, WNT ligands *(niche signals)*

**Immune-derived cells (ICs)**	Ovarian interstitium (stroma), around follicles, corpus luteum, blood vessels; concentrated in areas of active tissue remodeling	**Macrophages**<br>- Tissue remodeling<br>- Clearance of apoptotic cells<br>- Ovulatory inflammation<br>- Modulate follicle atresia and repair<br>- Angiogenesis regulation<br>- Immune surveillance	- CD68<br>- CD163<br>- CD14<br>- CSF1R<br>- CX3CR1<br>- FCGR1A (CD64)<br>- CD11B	- CD163, MRC1 (CD206), CD209 *(alternatively activated/tissue-repair)*<br>- CCR2, LY6C *(inflammatory monocyte-derived macrophages; context-dependent)*	- CD68<br>- ADGRE1 (F4/80)<br>- CD163<br>- CD14<br>- CSF1R<br>- CX3CR1<br>- FCGR1 (CD64)<br>- CD11B	- CD163, MRC1 (CD206) *(repair/tissue-resident phenotypes)*<br>- CCR2, LY6C *(inflammatory monocyte-derived macrophages)*
		**Dendritic cells**<br>- Antigen presentation<br>- Orchestrate adaptive immune responses<br>- Modulate tolerance vs inflammation in ovulatory and pathological contexts	- CD11C<br>- HLA-DR (MHC II)<br>- CD1C<br>- FCER1A<br>- CLEC10A<br>- CD209<br>- CD141	- CD1C, FCER1A, CLEC10A *(inflammatory states)*<br>- CD141, CLEC9A, XCR1 *(cross-presentation; context-dependent)*	- CD11C<br>- H2-AB1/H2<br>- EB1 (MHC II)<br>- CD11B<br>- CD8A<br>- CD103	- CD11C, CD11B,<br>- CD8A, CD103, XCR1, CLEC9A *(context-dependent)*
		**T cells**<br>- Immune surveillance<br>- Modulate follicle atresia, luteal regression, and inflammation<br>- Regulatory vs effector functions in PCOS/POI	- CD3D/E/G (CD3 complex), - CD4<br>- CD8A/B<br>- FOXP3 (Treg)<br>- IL7R<br>- TRAC	- FOXP3, IL2RA (CD25) *(Treg; tolerance, pregnancy, PCOS/POI contexts)*<br>- IFNG, IL17A, GZMB, PRF1 *(effector Th1/Th17/CTL signatures)*	- CD3D/E/G<br>- CD4<br>- CD8A/B<br>- FOXP3<br>- IL7R<br>- TRAC	- FOXP3<br>- IL2RA (TREG)<br>- IFNG, IL17A, GZMB, PRF1 *(effector states)*
		**Mast cells (limited)**<br>- Release histamine, proteases, cytokines<br>- Modulate vascular permeability, inflammation, and fibrosis<br>- Implicated in PCOS/POI	- TPSAB1 (tryptase)<br>- CPA3<br>- KIT (CD117/c-KIT)<br>- MS4A2 (FcεRIβ)<br>- FLT3 (CD135)	- TPSAB1<br>- CPA3<br>- KIT (*higher in activated / expanded mast cell populations in fibrosis)*<br>- IL13, IL4, TGFB1 (*mast cell–associated cytokines; context-dependent)*	- TPSAB1<br>- TPSB2<br>- CPA3<br>- KIT<br>- MS4A2<br>- FLT3	- TPSAB1, CPA3, KIT *(activated mast cells in inflammation/fibrosis)*<br>- IL13, IL4, TGFB1

**Large luteal cells (LLCs)**	Corpus luteum	- Progesterone production	- STAR<br>- CYP11A1<br>- HSD3B1/2<br>- NR5A1<br>- PGR<br>- CYP19A1<br>- LHCGR *(lower than in SLCs)*	- ANG<br>- GPR183<br>- INHBA<br>- PTGS2 (COX 2)<br>- PTGER1	- STAR<br>- CYP11A1<br>-HSD3B1/2<br>- NR5A1<br>- PGR<br>- CYP19A1	- SFRP4<br>- HSD17B7<br>- PTGS2,<br>- VEGFA<br>- ANGPT2<br>- BMP2<br>- WNT4
**Small luteal cells (SLCs)**	Corpus luteum	- Response to LH or CG stimulation	- STAR<br>- CYP11A1<br>- HSD3B1/2<br>- NR5A1<br>- PGR<br>- CYP19A1<br>- LHCGR<br>- LDLR<br>- SCARB1	- LHCGR<br>- LDLR<br>- PIM1<br>- SCARB1<br>- CYP11A1<br>- JUN<br>- FOS<br>- PTGS2	- STAR<br>- CYP11A1<br>- HSD3B1/2<br>- NR5A1<br>- LHCGR<br>- PGR<br>- LDLR<br>- SCARB1	- LHCGR<br>- SCARB1<br>- LDLR<br>- PTGS2<br>- VEGFA
**Ovarian endothelial cells (OECs)**	Blood and lymphatic vessels	- Establish of the entire vascular structure	- PECAM1<br>- CDH5<br>- EMCN<br>- VWF<br>- KDR<br>- FLT1	- ESM1<br>- PGF<br>- ANGPT2<br>- VEGFA-response genes<br>- VCAM1<br>- ICAM1	- PECAM1<br>- CDH5<br>- EMCN<br>- VWF<br>- KDR<br>- FLT1<br>- ESAM	- FLT1<br>- MMRN2<br>- ESM1<br>- VEGFA-response genes<br>- PROX1, LYVE1 and PDPN *(for lymphatic endothelium)*
**Ovarian surface epithelial cells (OSECs)**	Ovarian surface epithelium	- Protection of the ovary<br>- Ovulation-induced rupture and repair	- KRT7<br>- KRT8<br>- KRT18,<br>- KRT19<br>- MUC1<br>- EPCAM<br>- CDH2	- KRT17<br>- KRT5<br>- KRT14<br>-ITGA6, NGFR, MSLN *(wound-repair and estrogen-responsive genes)*	- UPK3B<br>- KRT7<br>- KRT8<br>- KRT18<br>- KRT19<br>- KRT7<br>- EPCAM	- KRT17, KRT5, KRT14, LGR5, *(wound-repair genes and ovulation-responsive epithelial markers)*
